# *S*-adenosylmethionine blocks osteosarcoma cells proliferation and invasion *in vitro* and tumor metastasis *in vivo*: therapeutic and diagnostic clinical applications

**DOI:** 10.1002/cam4.386

**Published:** 2015-01-26

**Authors:** Surabhi Parashar, David Cheishvili, Ani Arakelian, Zahid Hussain, Imrana Tanvir, Haseeb Ahmed Khan, Moshe Szyf, Shafaat A Rabbani

**Affiliations:** 1 Department of Medicine, McGill University Health CentreMontreal, QC, Canada; 2 Department of Pharmacology and Therapeutics, McGill University Health CentreMontreal, QC, Canada; 3 Fatima Memorial Hospital SystemLahore, Pakistan

**Keywords:** DNA methylation, gene expression, osteosarcoma, patients, signaling pathways, tumor progression

## Abstract

Osteosarcoma (OS) is an aggressive and highly metastatic form of primary bone cancer affecting young children and adults. Previous studies have shown that hypomethylation of critical genes is driving metastasis. Here, we examine whether hypermethylation treatment can block OS growth and pulmonary metastasis. Human OS cells LM-7 and MG-63 were treated with the ubiquitous methyl donor *S*-adenosylmethionine (SAM) or its inactive analog *S*-adenosylhomocystine (SAH) as control. Treatment with SAM resulted in a dose-dependent inhibition of tumor cell proliferation, invasion, cell migration, and cell cycle characteristics. Inoculation of cells treated with 150 *μ*mol/L SAM for 6 days into tibia or via intravenous route into Fox Chase severe combined immune deficient (SCID) mice resulted in the development of significantly smaller skeletal lesions and a marked reduction in pulmonary metastasis as compared to control groups. Epigenome wide association studies (EWAS) showed differential methylation of several genes involved in OS progression and prominent signaling pathways implicated in bone formation, wound healing, and tumor progression in SAM-treated LM-7 cells. Real-time polymerase chain reaction (qPCR) analysis confirmed that SAM treatment blocked the expression of several prometastatic genes and additional genes identified by EWAS analysis. Immunohistochemical analysis of normal human bone and tissue array from OS patients showed significantly high levels of expression of one of the identified gene platelet-derived growth factor alpha (PDGFA). These studies provide a possible mechanism for the role of DNA demethylation in the development and metastasis of OS to provide a rationale for the use of hypermethylation therapy for OS patients and identify new targets for monitoring OS development and progression.

## Introduction

Osteosarcoma (OS) is third most common childhood cancer affecting long bones accounting for 20% of all bone cancers 1,2. Late stage OS tumors are known to cause lung metastasis resulting in the high morbidity and mortality. Late stage disease is highly aggressive with 5-year event-free survival in 60–70% patients 3,4. While recent advances in neoadjuvant chemotherapy and surgery has improved the long-term survival rates of patients without metastatic disease, patients who exhibit metastasis continue to respond poorly to chemotherapy and have poor prognosis 5–8. This poor response to therapy is also associated with a high incidence of drug toxicity and efforts to change chemotherapeutic regimen has yielded limited success with no improvement in outcome 9. Therefore, it is crucial to understand the molecular mechanism of tumor metastasis for early diagnosis, predict prognosis, and identify new targets for the development of more effective therapeutic strategies.

OS is a rare tumor which is often difficult to classify. The primary malignant tumor is characterized by genetic instability and complex karyotypes 10. Various mutations, deletions, translocations, and amplifications aid to tumor development 10. Mostly alterations in two prominent tumor suppressor genes *TP53* and *RB1* are associated with tumorigenic activity 11. The p53 and retinoblastoma protein pathways are known for controlling apoptosis, DNA repair, and cell cycle regulation. However, epigenetic mechanisms are also known to contribute to the tumor development process in various types of cancers including OS 12–14. These epigenetic modifications mainly involve DNA methylation, histone modifications, and chromatin remodeling 15. The epigenome can regulate the alterations of DNA and associated proteins without affecting the original DNA sequence 16. One of the fundamental epigenetic modifications is the methylation of cytosine residues in CpG dinucleotides. Atypical methylation patterns have been observed in majority of cancers, which result in the inactivation of tumor suppressor pathways 17. Additionally, extensive hypomethylation of tumor-promoting genes is also described to enhance the overall process of oncogenesis. A recent delineation of the landscape of DNA methylation in liver cancer revealed widespread hypomethylation of promoters of genes involved in migration and invasion including several classic prometastatic genes 18. Hypermethylation of DNA caused by DNA methyltransferase enzymes (DNMTs) and histone acetylation by histone acetyltransferase (HAT) and histone deacetylase (HDAC) has been the prime focus of the epigenetic studies in the recent past 19. Drugs that target DNMTs and HDAC are under clinical trials for treatment of solid tumors and have already been approved for hematological malignancies 19. However, inhibition of DNA methylation could also result in activation of prometastatic genes and aggravate cancer metastasis 20,21. We therefore proposed that inhibition of demethylation of prometastatic genes could serve as a strategy to block cancer metastasis 22.

SAM is a common cosubstrate involved in methyl group transfer reactions 23. We have previously shown that SAM treatment causes hypermethylation of urokinase type plasminogen activator (uPA) in breast cancer cells and the knock down of methyl DNA-binding protein 2 resulting in silencing of the uPA gene by reverting the hypomethylated state of this gene in breast and prostate cancer cells 24,25. We have also previously shown that SAM could inhibit the proinvasive effects of the DNA methylation inhibitor Vidaza (5-azacytidine) on noninvasive breast cancer cells 25. We therefore tested in the present study whether methylating agent SAM would be effective in suppressing metastasis in OS in vitro and in vivo using well-established models of OS by effecting key signaling pathways involved in bone remodeling and tumor progression. Since methylation of tumor suppressor genes could stimulate cancer growth, we also determined whether SAM would not exhibit such an adverse effect. Our data show that SAM is effective in inhibiting both invasiveness and tumor growth. These data have important implications on therapy of metastatic OS.

## Materials and Methods

### Cell culture

Human OS cells LM-7 and MG-63 were obtained from the American Type Culture Collection and maintained in MEM with 10% fetal bovine serum, 2 mmol/L l-glutamine, and 100 units/mL penicillin sulfate/streptomycin sulfate. Cells were incubated with different doses of SAM or SAH (New England Biolabs, Mississauga, ON, Canada) as described previously 25.

### Cell proliferation invasion and wounding assay

LM-7 and MG-63 cells were plated in duplicates at a density of 9 × 10^5^ and 5 × 10^5^ cells, respectively, in 10 mL of culture media in plates. The effect of two different doses of SAM (75.0 and 150.0 *μ*mol/L) was evaluated. The invasive capacity of LM-7 and MG-63 cells was examined using two-compartment Boyden chamber Matrigel invasion assay (Costar Transwell, Corning Corporation, Sigma-Aldrich, Oakville, ON, Canada) following treatment with SAH or SAM for 6 days as described previously 25.

For wound healing analysis, cells LM-7 and MG-63 cells were treated with SAH or SAM (75 and 150 *μ*mol/L) for 6 days in the presence of 10% fetal bovine serum (FBS). Cells were then plated in six-well plates to form a monolayer and then wounded manually with a sterile 1000 *μ*L pipette tip in the center of each well. Cells were grown in the presence of 2% FBS and migrating cells where photographed at different time points. Analysis and quantification was carried out using Image Pro-Plus software and calculated as percentage wound healing using the equation, % wound healing = [1 − (wound area at *T*x h/wound area at *T*_0_)], where *T*x is the respective time point and *T*_0_ is the time immediately after wounding. These experiments were repeated twice in duplicates.

### Colony formation assay and cell cycle analysis

LM-7 and MG-63 cells at a density of 5000 cells per well previously treated with SAH (150 *μ*mol/L) or SAM (75 and 150 *μ*mol/L) for 6 days were seeded in triplicates into six-well Petri dishes in the presence of 4 mL of culture medium containing 1.5% agar solution at 37°C. Medium was changed every 48 h. After 14 days post plating, the number of colonies containing more than 100 cells was recorded.

For cell cycle analysis, LM-7 cells were treated with SAH or SAM (75 and 150 *μ*mol/L) every 48 h for 6 days and were fixed by adding 70% of ice-cold ethanol. Fixed cells were washed with phosphate-buffered saline (PBS) and then treated with 1 U of DNase-free RNase and stained with 0.05 mg of propidium iodide for 1 h. Cell cycle analysis was performed on a FACS Calibur (BD Biosciences, Mississauga, Canada) machine. Results were analyzed further using the FlowJo Software.

### Quantitative real-time polymerase chain reaction

For quantitative real-time polymerase chain reaction (qPCR) analysis, total cellular RNA from SAH- and SAM-treated LM-7 cells was extracted using TRIzol (Invitrogen Life Technologies, Burlington, ON, Canada) according to the manufacturer's protocol. Two micrograms of total RNA was used for reverse transcription (RT) reaction. Twenty-five nanograms of cDNA was used in a 20 *μ*L reaction with SYBR green mix, 0.8 *μ*mol/L forward and reverse primers. PCR was performed in an ABI StepOne Plus (Life Technologies, Burlington, Canada) with the following conditions: denaturation at 95°C for 10 min; amplification at 95°C for 15 sec, annealing temperature 1 min, for 40 cycles.

### Illumina Methylation 450K analysis

LM-7 cells were treated with vehicle or 150 *μ*mol/L of SAM for 6 days. Genomic DNA was quantified using Picogreen protocol (Quant-iT™ PicoGreen® dsDNA Products, Invitrogen, P-7589) and read on a SpctraMAX GeminiXS Spectrophotometer. Bisulfite conversion of 500 ng of genomic DNA was performed using the EZ-96 DNA Methylation-GOLD Kit (Zymo Research, Irvine, CA). The Illumina Methylation 450K kit (San Diego, California, USA) was used for the microarray experiment as described by the manufacturer's protocol, except that 8 *μ*L of bisulfite converted template was utilized to initiate the amplification step. The Illumina hybridization oven was used for incubating amplified DNA (37°C) and for BeadChips hybridization (48°C). A Hybex incubator was used for fragmentation (37°C) and denaturation (95°C) steps. The X-stain step was carried out in a Tecan Freedom evo robot with a Te-Flow module. Arrays were scanned in Illumina iScan Reader. Data analysis was performed with the Methylation module (version 1.9.0) of the GenomeStudio software (Illumina; version 2011.1) using HumanMethylation450_15017482_v1.2. bpm manifest. Statistical threshold was set at a false discovery rate of >0.05, differential score (statistical power) of >0.13, and delta beta (differential methylation) between the groups was set at >0.15.

### Immunohistochemical analysis of normal bone and clinical biopsies from OS patients

Tissue microarray slides for OS cases were obtained from US Biomax Inc. (Rockville, MD), whereas all normal cases were from iliac crest. Rabbit polyclonal antibodies for EXOC7 and PCGF3 (Abcam, Toronto, ON, Canada) were used at 1:10, 1:10, and 1:1000 dilution, respectively. Rabbit polyclonal antibody to PDGF AA (Abcam) was used as primary antibody at 1:1000 dilution. Heat-mediated antigen retrieval was performed by Tris/EDTA pH 9.0 buffer, EnvisionTM FLEX Target Retrieval Solution (Dako, Burlington, ON, Canada) at 1:50 dilution; and phosphate buffer containing hydrogen peroxide, 15 mmol/L NaN3 and detergent, EnvisionTM FLEX Peroxidase Blocking Reagent (Dako) was used as blocking reagent. Dextran coupled with peroxidase molecules and goat secondary antibody molecules against rabbit immunoglobulins in buffered solution containing stabilizing protein and preservative, EnvisionTM FLEX/HRP (Dako) was used as secondary antibody for 30 minutes. 3,3′-diaminobenzidine tetrachloride, EnvisionTM FLEX DAB+ Chromogen (Dako) and buffered solution containing hydrogen peroxide and preservative, EnvisionTM FLEX Substrate buffer (Dako) were added. The slides were counterstained with hematoxylin (1a Harris hematoxylin solution by MERCK KGaA, Darmstadt, Germany). Sections were washed twice for 10 min in Tris-buffered saline solution containing Tween 20, pH 7.6 (EnvisionTM FLEX Wash Buffer (Dako) at 1:20 dilution after every step during the procedure. Slides were mounted with DPX (MERCK, KGaA).

Stained slides were scored for proportion and intensity of staining in cells by two pathologists. Staining intensity was assessed as negative, mild, moderate, or strong. Percentage of positive cells showing different intensity staining patterns were noted, and then rounded off to the nearest 10th percentage. Percentage of cells showing mild intensity were given score 1, percentage of cells showing moderate intensity were given score 2, and those with strong intensity staining were given score 3 26,27. A total score was obtained by adding the products of these different intensity scores as follows. Total Score = (percentage of cells with mild intensity staining × 1) +(percentage of cells with moderate intensity staining × 2) + (percentage of cells with strong intensity staining × 3).

### Animal protocols

For in vivo studies, LM-7 cells were treated with SAH or SAM (150 *μ*mol/L) for 6 days in MEM + 10% FBS. At the end of the treatment, cells were harvested in sterile saline. Six-week-old male Fox Chase severe combined immune deficient (SCID) mice, obtained from Charles River, St-Constant, QC, Canada, were anesthetized using a cocktail of ketamine (50 mg/kg), xylazine (5 mg/kg), and acepromazine (1 mg/kg) intramuscularly. LM-7 cells viability was confirmed by Trypan blue assay and cells were inoculated at 2 × 10^5^ cells per mouse in 40 *μ*L saline with a 27-gauge needle into the left tibia using a drilling motion. The mice were monitored weekly for tumor burden. On week 4, a digital radiography of hind limbs of all animals was done using a Faxitron X-ray machine (Faxitron X-ray Corp., Lincolnshire, IL) to monitor the development of skeletal lesions. The mice were then euthanized, and the left tibias were collected and fixed in 10% buffered formalin solution for 24 h. The X-ray scoring method is described as follows: no lesions or minor changes, small lesions, significant lesions (minor peripheral margin breaks, 1–10% of bone surface disrupted), and significant lesions (major peripheral margin breaks, >10% of bone surface broken) rating 0–4, respectively 28–32.

In lung metastasis studies, LM-7 cells treated with 150 *μ*mol/L of SAH or SAM were inoculated in 6-week-old female BALB/c nude mice, tumor formation and pulmonary metastasis was monitored for a period of 14 weeks 33,34. Control and experimental animals were sacrificed at the end of this period and lungs were harvested and fixed. Metastatic nodules were counted on surfaces of all lung lobes and the number recorded as the number of lung metastases for each tumor-bearing animal. All the experimental animal protocols were in accordance with the McGill University Animal Care Committee guidelines.

### Statistical analysis

Results were analyzed as the mean ± SEM, and comparisons of the experimental data were analyzed by an independent two-sample *t*-test at *P* < 0.05 level of significance.

## Results

### Effect of SAM on OS cells proliferation, invasion, and migration

**Figure 1:**
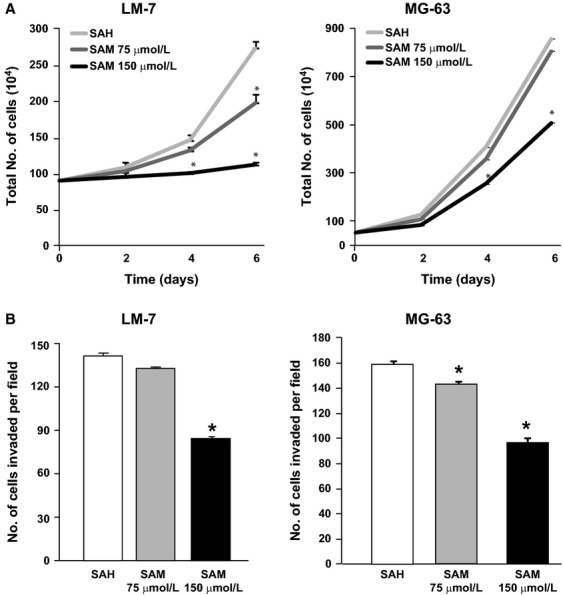
Effect of *S*-adenosylmethionine (SAM) on osteosarcoma (OS) cells proliferation and invasion *in vitro*. Human OS cancer cells LM-7 and MG-63 were plated in 10 mL plates and treated with 150 *μ*mol/L of *S*-adenosylhomocystine as control (SAH) or two doses (75 and 150 *μ*mol/L) of SAM. Cell growth rate was determined in each group by trypsinization and counting the number of cells by Coulter counter as described in Materials and Methods section (A). LM-7 and MG-63 cells invasive capacity was evaluated by using a Boyden chamber Matrigel invasion assay. After 18 h of SAM (75 and 150 *μ*mol/L) treatment, the invaded cells were fixed, stained, and 10 random fields were counted. Number of cells invading is shown as bar diagram ± SEM (B) as described in Materials and Methods section. Results are presented as the mean ± SEM of two different experiments in duplicate from control and experimental cells. Significant differences from the control (SAH) is represented by an asterisk (*P* < 0.05).

Methylation of tumor suppressor genes could result in increased growth rate, which might counteract any antimetastatic property of SAM. We therefore first determined whether SAM treatment would result in adverse increase in cancer cell growth rate. We examined the effect of SAH and SAM treatment on two invasive human OS cell lines LM-7 and MG-63. Treatment of LM-7 and MG-63 with 75 and 150 *μ*mol/L dose of SAM for 6 days resulted in significant inhibition of LM-7 and MG-63 cell proliferation as compared to control cells treated with similar doses of SAH (Fig.1A).

We then determined whether SAM treatment affects the invasive potential of OS cells using Boyden chamber Matrigel invasion assay. Treatment of LM-7 and MG-63 cell lines with different doses (75 and 150 *μ*mol/L) of SAM reduced tumor cells invasion in a dose-dependent manner (Fig.1B). In order to rule out the possible confounding antiproliferative effects of SAM as shown in panel A, we counted the tumor cells in both upper and lower part of Boyden chamber. Results from this analysis showed similar number of tumor cells during this treatment demonstrating that the observed anti-invasive effects are not due to the ability of SAM to alter cell proliferation.

**Figure 2:**
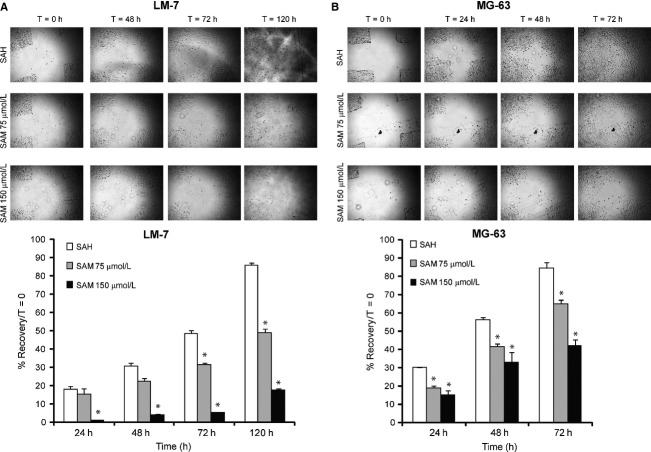
Effect of *S*-adenosylmethionine (SAM) on osteosarcoma (OS) cells migration in vitro. Wound healing assay was carried out by seeding LM-7 (A) and MG-63 (B) cells in six-well plates and allowing them to grow as a monolayer and making a wound as described in Materials and Methods section. These cells were treated with 150 *μ*mol/L *S*-adenosylhomocystine as control (SAH) or two different doses of SAM (75 and 150 *μ*mol/L) containing 2% fetal bovine serum and migrating cells were photographed at different time points. Percent wound healing was recorded at different time points, and percentage of wound healing with respect to *T*_0_ was calculated using the equation described in Materials and Methods section. Results are presented as the mean ± SEM of two different experiments in duplicate from control and experimental cells. Significant differences from the control (SAH) is represented by an asterisk (*P *<* *0.05).

The effect of SAM on cell migration was analyzed by wound healing assay using LM-7 and MG-63 cell lines. A significant reduction in wound healing (%) was observed in SAM-treated (75 and 150 *μ*mol/L) LM-7 cells compared with SAH-treated control cells at 48, 72, and 120 h and MG-63 cells at 24, 48, and 72 h after wounding (Fig.2). At 120 and 72 h, 150 *μ*mol/L of SAM was most effective in blocking cell migration in LM-7 and MG-63, respectively.

### Effect of SAM on colony formation and cell cycle

**Figure 3:**
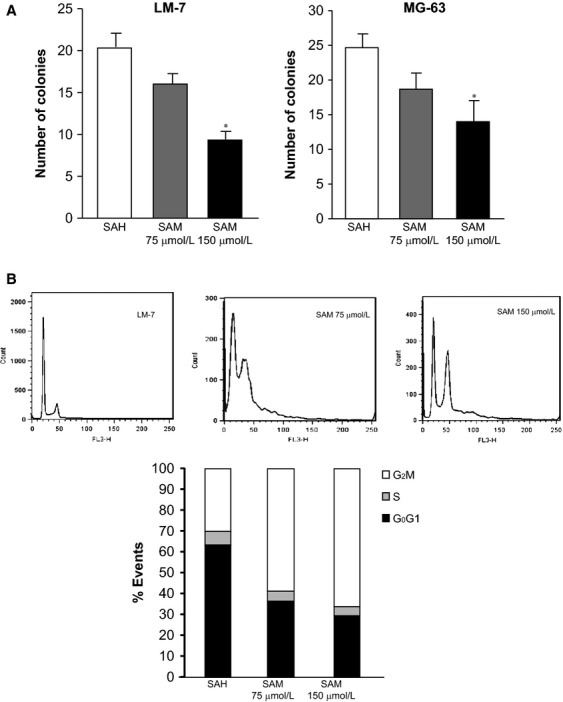
Effect of *S*-adenosylmethionine (SAM) on osteosarcoma (OS) cells colony formation and cycle kinetics in vitro. LM-7 and MG-63 cells were plated onto soft agar for anchorage independent growth in the presence of 150 *μ*mol/L *S*-adenosylhomocystine as control (SAH) or SAM (75 and 150 *μ*mol/L). Number of colonies was counted as described in Materials and Methods section (A). LM-7 and MG-63 cells were treated with 150 *μ*mol/L of SAH as control (SAH) or SAM (75 and 150 *μ*mol/L). Treated cells were then fixed and stained with propidium iodide. FACS analysis was performed as described in Materials and Methods section (B). Results are presented as the mean ± SEM of two different experiments in duplicate from control and experimental cells. Significant differences from the control (SAH) is represented by an asterisk (*P *<* *0.05).

Tumor cell's ability to form colonies in soft agar is an index of their aggressive potential. We therefore examined the effect of SAM on the number of colonies formed by LM-7 and MG-63 cells. Following treatment of these cells with (75 and 150 *μ*mol/L) of SAM, a significant and dose-dependent decrease in the number of colonies formed was observed compared to control (SAH-treated) group of cells (Fig.3A).

We then examined the effects of different doses (75 and 150 *μ*mol/L) of SAM on cell cycle kinetics to further confirm that SAM treatment would not result in silencing of tumor suppressor mechanisms and enhancement of cell cycle progression. FACS analysis of cell cycle distribution on control and SAM-treated cells showed a significant increase in the number of tumor cells in G_2_/M phase with simultaneous decrease in S phase in the SAM treatment group as compared to control group of cells (Fig.3B). Thus, not only does SAM accelerate the progression of the cell cycle as anticipated if it silenced tumor suppressor genes but also it inhibits progression through arresting cells at the G_2_/M phase of the cell cycle.

### Effect of SAM on OS metastasis in vivo

**Figure 4:**
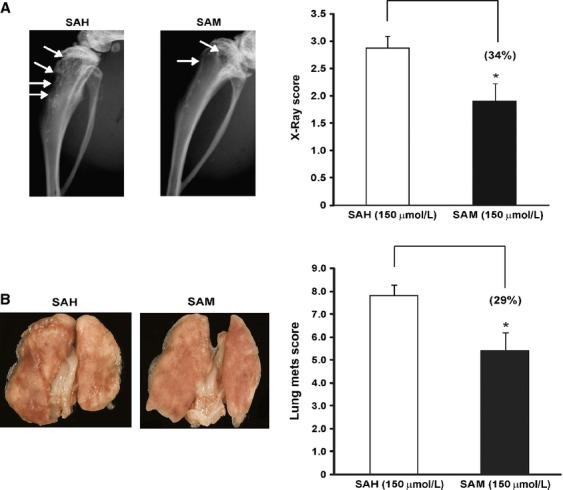
Effect of *S*-adenosylmethionine (SAM) on osteosarcoma (OS) skeletal lesions and lung metastasis in vivo. (A) Male Fox Chase severe combined immune deficient (SCID) mice were inoculated with (2 × 10^5^) LM-7 cells treated with 150 *μ*mol/L of *S*-adenosylhomocystine as control (SAH) or 150 *μ*mol/L of SAM for 7 days via intravenous route. Development of skeletal lesions was determined at weekly intervals by X-ray using Faxitron and lesion area was determined as described Materials and Methods section. Representative X-ray and lesion score of control and experimental animals at week 4 post tumor cell inoculation is shown. Skeletal lesions are highlighted by arrows. (B) Male Balb/c nude mice were inoculated with (2 × 10^5^) LM-7 cells treated with 150 *μ*mol/L of SAH as control (SAH) or 150 *μ*mol/L of SAM for 7 days and injected via tail vein. At week post tumor cells inoculation control and experimental animals were sacrificed and number lung metastasis was determined as described in Materials and Methods section. Photomicrographs of representative lungs in each group are shown. Result represents the mean ± SEM of ten animals in each group. Significant differences from control are represented by asterisks (*P *<* *0.05).

Next, we examined the effect of SAM on development and progression of skeletal lesions in our xenograft model of OS by using highly invasive LM-7 cells. Control and SAM (150 *μ*mol/L)-treated LM-7 cells were inoculated directly into the tibia of male Fox Chase mice as described in Materials and Methods section. Control animals developed skeletal lesions at week 8 which continued to increase in size and number of lesions over time. In contrast, animals treated with LM-7 cells treated with SAM exhibited reduced total skeletal lesion area (∼34%) represented as X-ray score as compared to the control group of animals inoculated with SAH at week 8 post tumor cell inoculation (Fig.4A).

Since lung metastasis is a common occurrence in OS, we next examined the effect of SAM treatment on the development of lung metastasis using our lung metastasis model as described in Materials and Methods section. Control animals inoculated with SAH-treated LM-7 cells developed large lung metastasis detected at the end of these studies 14 weeks post tumor cell inoculation. In contrast, experimental animals inoculated with SAM-treated LM-7 cells exhibited a marked decrease in number and size of lung metastasis (Fig.4B).

### Effects of SAM on epigenome wide methylation in OS

SAM is a global hypermethylating agent raising the concern that it will indiscriminately affect DNA methylation particularly methylating tumor suppressor genes, which could result in enhancing cancer cell growth. Although our cellular studies described in Figures1 and 3 demonstrated that SAM did not block tumor suppressor mechanisms, but rather enhanced tumor suppression it is nevertheless important to exclude the possibility that SAM increases methylation of tumor suppressor genes. We therefore performed an epigenome wide analysis of the changes in DNA methylation triggered by SAM using Illumina 450K bead arrays which provide a representative coverage of CGs at transcription start sites, 5′ regulatory regions, CG shores as well as in the gene bodies. DNA was isolated from LM-7 cells treated with 150 *μ*mol/L of SAH and SAM for 6 days. This dose and time period of treatment was found to be most effective in inhibiting tumor cell proliferation, invasion, and migration (Figs.1 and 2). Results from these studies presented in Table S1 which lists the statistically significant CGs whose methylation was altered in response to SAM treatment reveal that SAM has remarkably a very specific and particularly limited effect on the methylome. None of the known tumor suppressor genes altered their state of methylation in response to SAM treatment, while the sites that were hypermethylated were associated with genes that were known to play a key role in tumor growth and metastasis (Table S1). Ingenuity pathway analysis (IPA) showed that the hypermethylated genes are members of key intracellular signaling pathways that are known to be involved in OS growth and metastasis, but there were no genes in tumor suppressor pathways that seem to be affected (Table S2).

### Effect of SAM on the expression of OS-associated genes

**Figure 5:**
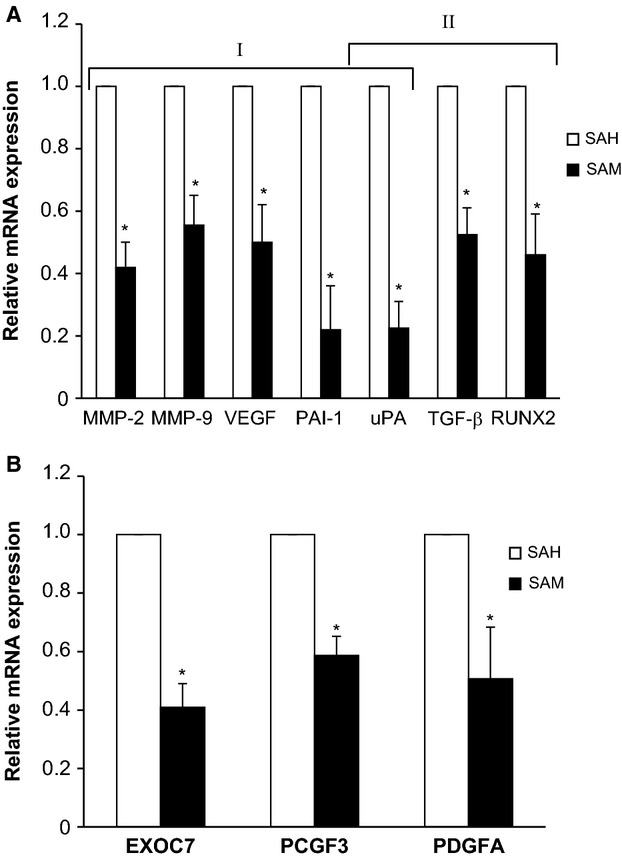
Effect of *S*-adenosylmethionine (SAM) on the expression of genes associated with osteosarcoma (OS) metastasis. LM-7 cells were treated with 150 *μ*mol/L of *S*-adenosylhomocystine as control (SAH) or with 150 *μ*mol/L of SAM for 7 days, and total cellular RNA was isolated with TRIzol. RNA from control and treatment groups were analyzed for the expression of genes involved in tumor progression and skeletal metastasis (A) and hypomethylated genes identified by Illumina analysis (B). Changes in the mRNA expression of the representative genes were determined by plotting the relative ratio against GAPDH which was used as loading control. Results are presented as the mean ± SEM of two different experiments in duplicate from control and experimental cells. Significant differences from the control (SAH) is represented by an asterisk (*P *<* *0.05).

**Figure 6:**
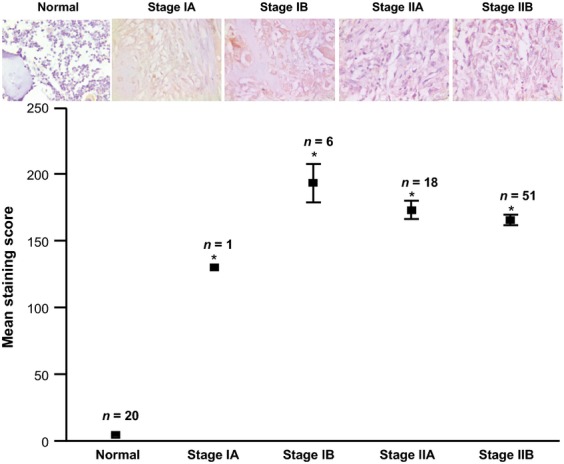
Immunohistochemical analysis for platelet-derived growth factor alpha (PDGFA) expression in normal bone and osteosarcoma patients. Tissue array obtained from of osteosarcoma patients of different stages (Ia, Ib, IIa, IIb) as defined by the American Cancer Society and normal bone (Normal) were stained with PDGFA-specific antibody and staining intensity was quantitated as described in Materials and Methods section. The staining intensity was calculated and a total mean staining score = (Percentage of cells with mild intensity staining × 1) + (Percentage of cells with moderate intensity staining × 2) + (Percentage of cells with strong intensity staining × 3) was calculated and represented in (lower panel). Representative images from normal bone and different stages of osteosarcoma are shown (upper panel). Significant differences from control are represented by asterisks (P < 0.05).

Due to the complex nature of OS progression several molecular pathways and genes are implicated in its growth and metastasis. In order to understand the antitumor effects of SAM, we first analyzed the expression of well-established genes which are known to alter tumor cell proliferation, invasion, and metastasis as well as genes that were hypermethylated by 150 *μ*mol/L 6 d SAM treatment as determined by the Illumina bead array analyses (Tables S1, S2). The qPCR results presented in Figure 6 show the analysis of RNA from control and 150 *μ*mol/L SAM-treated cells. SAM treatment reduced the expression of genes implicated in tumor cell invasion, metastasis, and angiogenesis such as *matrix metalloproteinase (MMP) 2 and 9*, *vascular endothelial growth factor (VEGF)*, *plasminogen activator inhibitor 1 (PAI-1)*, and *uPA*. Additionally, SAM treatment also markedly reduced the expression of transforming growth factor *β (TGF-β)* and *runt-related transcription factor 2 (RUNX2)* (Fig.5A).

We also selected three representative genes of Exocyst Complex Component 7 (EXOC7), Polycomb Group Ring Finger 3 (PCGF3), and Platelet-Derived Growth Factor Alpha (PDGFA) which were found to be hypermethylated following SAM treatment. These genes are involved with several intracellular signaling pathways that are known to affect tumor growth and metastasis 35,38. qPCR analysis of control and experimental LM-7 cells show that expression of these genes was markedly reduced following SAM treatment supporting the hypothesis that SAM triggered hypermethylation leads to silencing of several genes critical for metastasis (Fig.5B).

### Expression of new candidate genes in cancer and normal tissues

We determined the levels of expression of these genes (EXOC7, PCGF3, PDGFA) in normal bone and clinical biopsies from OS patients in a tissue array using commercially available antibodies as described in “Material and Methods” section. Results from OS array demonstrated a higher PDGFA expression in different stages of OS as compared to normal bone (*P* < 0.05). No significant differences in the level of expression of PDGFA were observed between different stages of OS (Fig.6). Antibodies against EXOC7 and PCGF3 showed a high nonspecific staining at multiple dilutions resulting in inconclusive results for immunohistochemistry (data not shown).

## Discussion

Aberrations in DNA methylation pattern is one of the hallmarks of cancer where by controlling the transcription of tumor suppressor and prometastatic genes it can regulate the multistep process of tumor progression 39. In the majority of studies, to date focus has been on understanding the hypermethylation of tumor suppressor genes and targeting these processes therapeutically, whereas little attention was paid to the potential role of hypomethylation of prometastatic genes. However, an increasing body of evidence suggests that hypomethylation of prometastatic genes could promote cancer metastasis. This points to the possibility that drugs that induce hypermethylation of prometastatic genes could serve as antimetastatic agents. We have previously shown that the ubiquitous methyl donor SAM can inhibit DNA demethylation in vitro and in vivo 24,25 and can lead to hypermethylation and silencing of prometastatic genes. SAM is a particularly attractive agent since it is a FDA-approved nutritional supplement with little documented toxicity.

In this study, we provide a proof of principle that SAM could act as an antimetastatic agent in OS. Toward these goals, we used two in vivo models of OS which allowed the evaluation of the effect of SAM in bone and in blocking distant metastasis (lungs). Combined with these models, we used several in vitro assays to determine the mechanism of these antitumor effects of SAM. The first concern with using a hypermethylating agent in cancer is that it will lead to silencing of tumor suppressor genes through increased DNA methylation and that such an effect will override its beneficial effects on inhibition of metastasis. Our results show that SAM treatment had a significant effect on reducing tumor cell proliferation and altering cell cycle kinetics by reducing the number of cells in S phase and arresting them at G_2_/M phase. This suggests that SAM does not inhibit tumor suppressor mechanisms; on the contrary, SAM triggers mechanisms that arrest cell growth and makes them susceptible to radio- and chemotherapy. As hypothesized, SAM inhibited invasion and migration and thus blocked basic mechanisms driving metastasis while avoiding silencing of tumor suppressor mechanisms. Although SAM reduced both proliferation and invasion, the effects of SAM on cell invasion were found to be independent of cell death or inhibition of proliferation as similar number of control (SAH) and experimental (SAM) treated tumor cells were observed in upper parts of Boyden (Fig.1). We then evaluated the effect of SAM on OS metastasis in vivo. Inoculation of SAM-treated cells exhibited a significantly reduced number of lung metastasis when injected via tail vein in vivo. In vivo SAM treatment did not increase cell proliferation as anticipated if tumor suppressor genes were silenced by this hypermethylating agent but resulted in inhibition of cell proliferation. The fact that transient treatment in vitro was sufficient to block invasion and growth in vivo without further treatment with SAM is consistent with the hypothesis that the “in vitro” treatment epigenetically “reprogrammed” the OS cells to become less invasive and tumorigenic. The ability of epigenetic drugs to “reprogram” cancer cells carries important therapeutic advantage. The specificity of these SAM-mediated effects was confirmed by simultaneous treatment with its inactive analog SAH which lacks the methyl group and does not cause hypermethylation and showed no effects on invasion and growth.

Although SAM is a global hypermethylating agent, the biological effects observed suggest specificity 40. A plausible mechanism for SAM action is that it results in coordinate silencing of critical genes for OS metastasis but does not silence tumor suppressor genes. In order to understand the underlying molecular mechanism mediating these significant in vitro and in vivo affects, we first examined the change in the levels of expression of genes implicated in tumor metastasis in general and skeletal metastasis in particular. PCR analysis of control (SAH) and experimental (SAM) treated LM-7 cells showed a marked inhibition in the expression of tumor-promoting genes (*MMP-2*, *MMP-9*, *VEGF*, *PAI-1*, and *uPA*) and genes (*uPA*, *TFG-β*, and *RUNX2*) which are known to promote the development and progression of skeletal metastasis. *MMP-2* and *MMP-9* are two key regulators of extracellular matrix (ECM) remodeling and play a crucial role in angiogenesis, migration of cancer cells and metastasis. *VEGF* is a major angiogenic growth factor 41. uPA and PAI-1 are integral components of plasminogen activator system and play important roles in ECM degradation and invasion of cancer cells 42,43. TGF-*β* and RUNX2 are involved in osteoblast differentiation and skeletal metastasis 43,44. TGF-*β* arrests cell cycle at G1 phase and initiates differentiation or apoptosis of normal cells; however, in metastatic cancer it is known to stimulate invasion and metastasis by up regulating the uPA mRNA and SMAD4 signaling 9,45. *RUNX2* is a gene which has a well-established role in bone biology and skeletal metastasis 46. Recently, it has been shown that increased residence of RUNX2 at mitotic chromosomes may reflect its epigenetic function in “bookmarking” of target genes in cancer cells 47. The fact that SAM targeted these genes provides a plausible mechanism for its anti-OS effects seen in our study.

The idea that SAM has a specific effect on OS that targets prometastatic genes for silencing but not tumor suppressor genes was supported by a methylome analysis of changes in DNA methylation in LM-7 triggered by SAM (Table S1). Remarkable in spite of the fact that it is a general methyl donor only a small number of genes were affected by SAM (Table S1), but they seem to particularly target critical pathways for metastasis and tumor growth (Table S2). Ingenuity pathway analysis of these genes that became differentially methylated are involved in critical signaling pathways that were known to play a role in tumorigenesis but none of the known tumor suppressor genes that are hypermethylated in cancer. Following IPA analysis, we selected three genes that are hypermethylated by SAM treatment (*EXOC7*, *PCGF3*, *PDGFA*) which are implicated in several key intracellular signaling pathways, regulation of gene transcription, and tumorogenesis as shown in Table S1. We then determined the change in the levels of expression of these candidate genes (*EXOC7*, *PCGF3*, and *PDGFA*) in OS cells following treatment with SAM. Experimental cells treated with SAM showed a marked suppression in the expression of these genes as determined by qPCR analysis.

Using immunohistochemcial analysis, we determined the significance of identified genes (*EXOC7*, *PCGF3*, *PDGFA*) in the OS development and progression. Toward these goals we used commercially available OS tissue array and normal bone from our institution. Commercially available antibodies against *EXOC7* and *PCGF3* showed high nonspecific staining at multiple dilutions and results from these studies are not shown. However, antibody against PDGFA showed specific staining of bone cells. Results of this analysis as shown in Figure6 show low levels of PDGFA expression normal bone samples. In contrast, PDGFA expression was markedly high in OS patients. While these results clearly showed the induction of PDGFA in OS, limited number of samples from early stages (Ia, Ib) restricted our ability to establish a correlation with disease progression. These results are particularly significant as PDGFA is upregulated in several cancers due to its ability to alter cell proliferation, differentiation, angiogenesis, and metastasis 38,48,49.

Collectively, these results provide support that SAM can serve as a viable and attractive anticancer agent which blocks various tumor-promoting genes and signaling pathways. Our studies identify OS “signature” candidate genes, which are hypomethylated in OS and may serve as efficient biomarkers for diagnosis and prognosis of OS patients. Since SAM is already being used in oral formulation, it can provide beneficial effects in both preventive and therapeutic settings using improved and stable forms of SAM. Results from these studies also provide new therapeutic opportunities where methylation therapy alone or in combination with various therapeutic strategies currently under development to target genes which we have identified like uPA and its receptor to elicit strong synergistic effects to significantly reduce morbidity and mortality in cancer patients in general and those with OS in particular.

## Supporting Information

**Table S1.** Differentially methylated genes and their functions.

**Table S2.** Ingenuity pathway analysis of hypermethylated genes in SAM-treated LM-7 cells.
