# Supplementary material for: S-adenosylmethionine blocks osteosarcoma cells proliferation and invasion in vitro and tumor metastasis in vivo: therapeutic and diagnostic clinical applications
Source: Cancer Med. 2015 Jan 26;4(5):732–44. doi: 10.1002/cam4.386 (PMC4430266; doi:10.1002/cam4.386)
Supplement: Supplementary file 1 — Table S1. Differentially methylated genes and their functions. [file cam40004-0732-sd1.pdf]

Table 1. Differentially methylated genes and their functions

UCSC_REFGENE_ NAME	Function	UCSC_REFGENE_ ACCESSION	TargetID	UCSC_REFGENE_ GROUP	LM7_SAM. Delta Beta	LM7_SAM. DiffScore
EXOC7	Member of exocyst complex, vesicular trafficking	NM_001145298;NM_001145297; NM_015219;NR_028133;NM_001145299; NM_001013839	cg26287080	Body	0.29	113.7
			cg09973371		0.17	83.6
			cg03655023		0.19	79.7
PCGF3	Member of polycomb group, PcGPRC1 complex-chromatin remodeling	NM_006315	cg03271827	5'UTR	0.19	74.6
NXN	Nucleoredoxin, negative regulator of Wnt signaling pathway	NM_022463	cg05176970	Body	0.16	57.8
PDGFA	Growth factor, embryonic development, cell proliferation, migration chemotaxis	NM_002607;NM_033023	cg14496282	Body	0.16	32.3
PRKAR1B	Regulatory subunit of the cAMP-dependent protein kinases	NM_001164759;NM_001164762; NM_001164758;NM_001164760; NM_001164761;NM_002735	cg13558627	Body	0.12	31.4
			cg01635128		0.15	29.6
PTPRN2	Development of nervous system and pancreatic endocrine cells	NM_002847;NM_130842;NM_130843	cg00859877	Body	0.14	25.5
			cg08284447		0.11	25.2
ERICH1	Glutamate-rich protein 1	NM_207332	cg11416102	Body	0.13	25.2
INS-IGF2;IGF2AS; IGF2;	Insulin-like growth factor, potent mitogen, exhibits osteogenic effects	NR_003512;NR_028044; NM_001127598;NM_001007139; NR_028043;NM_000612	cg04072545	Body;TSS1500;5'UTR;	0.09	21.9
			cg14317384		0.16	20.0
			cg05146307		0.10	17.7

PRKAR1B	Regulatory subunit of the cAMP-dependent protein kinases	NM_001164762;NM_002735; NM_001164759;NM_001164761; NM_001164760;NM_001164758	cg22433798	3'UTR	0.15	16.4
CTSH	Cathepsin H, degradation of proteins in lysosomes	NM_004390;NM_148979	cg12604181	1stExon;5'UTR	0.13	15.7
			cg05546241		-0.12	-15.5
ERICH1	Glutamate-rich protein 1	NM_207332	cg23474407	Body	-0.17	-19.6
			cg25247689		-0.17	-21.4
FRMD4B	Member of GRP1 signaling complex, recruited in response to insulin receptor signaling	NM_015123	cg10178228	TSS200	-0.16	-24.6
PTPN14	Protein tyrosine phosphatase, lymphangiogenesis, regulates TGF-β, tumor suppressor	NM_005401	cg10952190	TSS1500	-0.15	-37.7
HOXA11	Transcription factor, part of developmental regulatory system	NM_005523	cg27309564	Body	-0.14	-41.8
			cg18430555		-0.18	-72.4
LOC442459		NR_024608	cg03789606	Body	-0.21	-76.6
			cg16578226		-0.29	-163.9
