# Supplementary material for: S-adenosylmethionine blocks osteosarcoma cells proliferation and invasion in vitro and tumor metastasis in vivo: therapeutic and diagnostic clinical applications
Source: Cancer Med. 2015 Jan 26;4(5):732–44. doi: 10.1002/cam4.386 (PMC4430266; doi:10.1002/cam4.386)
Supplement: Supplementary file 2 — Table S2. Ingenuity pathway analysis of hypermethylated genes in SAM-treated LM-7 cells. [file cam40004-0732-sd2.pdf]

Table 2 Ingenuity pathway analysis of hypermethylated genes in SAM treated LM7 cells

Ingenuity Canonical Pathways	p-value	Ratio	Molecules
Glioma Signaling	8.11E-04	1.77E-02	IGF2,PDGFA
Glioblastoma Multiforme Signaling	1.93E-03	1.19E-02	IGF2,PDGFA
Thioredoxin Pathway	2.76E-03	1.25E-01	NXN
Vitamin-C Transport	6.43E-03	4.55E-02	NXN
Sonic Hedgehog Signaling	1.37E-02	2.86E-02	PRKAR1B
Axonal Guidance Signaling	1.54E-02	4.14E-03	PDGFA,PRKAR1B
Netrin Signaling	1.78E-02	1.72E-02	PRKAR1B
Neuroprotective Role of THOP1 in Alzheimer's Disease	1.83E-02	1.82E-02	PRKAR1B
Amyloid Processing	2.33E-02	1.64E-02	PRKAR1B
Phototransduction Pathway	2.42E-02	1.49E-02	PRKAR1B
PXR/RXR Activation	3.05E-02	1.09E-02	PRKAR1B
Macropinocytosis Signaling	3.09E-02	1.3E-02	PDGFA
Growth Hormone Signaling	3.13E-02	1.28E-02	IGF2
Melatonin Signaling	3.18E-02	1.23E-02	PRKAR1B
Leptin Signaling in Obesity	3.40E-02	1.18E-02	PRKAR1B
BMP signaling pathway	3.40E-02	1.18E-02	PRKAR1B
Dopamine Receptor Signaling	3.45E-02	1.04E-02	PRKAR1B
PDGF Signaling	3.49E-02	1.16E-02	PDGFA
VDR/RXR Activation	3.54E-02	1.14E-02	PDGFA
Melanocyte Development and Pigmentation Signaling	3.89E-02	1.05E-02	PRKAR1B
α-Adrenergic Signaling	3.89E-02	9.17E-03	PRKAR1B
CDK5 Signaling	3.98E-02	1.03E-02	PRKAR1B
PAK Signaling	3.98E-02	9.09E-03	PDGFA
G Beta Gamma Signaling	4.03E-02	8.26E-03	PRKAR1B
IL-1 Signaling	4.20E-02	9.17E-03	PRKAR1B
PPAR Signaling	4.25E-02	9.35E-03	PDGFA
Antioxidant Action of Vitamin C	4.34E-02	9.09E-03	NXN
IGF-1 Signaling	4.38E-02	9.35E-03	PRKAR1B
Neuropathic Pain Signaling In Dorsal Horn Neurons	4.52E-02	9.17E-03	PRKAR1B
Nitric Oxide Signaling in the Cardiovascular System	4.56E-02	8E-03	PRKAR1B
Renin-Angiotensin Signaling	4.91E-02	7.94E-03	PRKAR1B
Sphingosine-1-phosphate Signaling	4.96E-02	8.13E-03	PDGFA
Androgen Signaling	5.04E-02	6.9E-03	PRKAR1B
Gαs Signaling	5.04E-02	8E-03	PRKAR1B
Corticotropin Releasing Hormone Signaling	5.09E-02	6.9E-03	PRKAR1B
Sperm Motility	5.31E-02	6.99E-03	PRKAR1B
Synaptic Long Term Potentiation	5.40E-02	7.69E-03	PRKAR1B
Atherosclerosis Signaling	5.44E-02	7.25E-03	PDGFA
P2Y Purigenic Receptor Signaling Pathway	5.48E-02	6.94E-03	PRKAR1B
Gαi Signaling	5.48E-02	7.41E-03	PRKAR1B
Cellular Effects of Sildenafil (Viagra)	5.79E-02	6.45E-03	PRKAR1B
GNRH Signaling	5.83E-02	6.54E-03	PRKAR1B
eNOS Signaling	5.88E-02	6.45E-03	PRKAR1B
Insulin Receptor Signaling	5.92E-02	6.71E-03	PRKAR1B
Ovarian Cancer Signaling	5.97E-02	6.58E-03	PRKAR1B
Human Embryonic Stem Cell Pluripotency	6.01E-02	6.21E-03	PDGFA
AMPK Signaling	6.01E-02	5.56E-03	PRKAR1B
Cardiac β-adrenergic Signaling	6.05E-02	6.33E-03	PRKAR1B
Relaxin Signaling	6.14E-02	6.1E-03	PRKAR1B
Hepatic Cholestasis	6.23E-02	5.46E-03	PRKAR1B
Hepatic Fibrosis / Hepatic Stellate Cell Activation	6.23E-02	6.45E-03	PDGFA

Hepatic Fibrosis / Hepatic Stellate Cell Activation	6.23E-02	6.45E-03	PDGFA
Tight Junction Signaling	6.83E-02	5.99E-03	PRKAR1B
Gap Junction Signaling	6.96E-02	5.52E-03	PRKAR1B
Cdc42 Signaling	7.18E-02	5.38E-03	EXOC7
Dopamine-DARPP32 Feedback in cAMP Signaling	7.27E-02	5.35E-03	PRKAR1B
RAR Activation	7.74E-02	5.24E-03	PRKAR1B
CREB Signaling in Neurons	7.78E-02	4.83E-03	PRKAR1B
Ephrin Receptor Signaling	7.78E-02	4.76E-03	PDGFA
PPARα/RXRα Activation	7.83E-02	5.03E-03	PRKAR1B
Sertoli Cell-Sertoli Cell Junction Signaling	7.87E-02	5.05E-03	PRKAR1B
Calcium Signaling	8.00E-02	4.61E-03	PRKAR1B
Role of NFAT in Cardiac Hypertrophy	8.08E-02	4.78E-03	PRKAR1B
Clathrin-mediated Endocytosis Signaling	8.21E-02	5.05E-03	PDGFA
ERK/MAPK Signaling	8.34E-02	4.74E-03	PRKAR1B
Breast Cancer Regulation by Stathmin1	8.55E-02	4.67E-03	PRKAR1B
Actin Cytoskeleton Signaling	9.44E-02	4.13E-03	PDGFA
cAMP-mediated signaling	9.70E-02	4.42E-03	PRKAR1B
Cardiac Hypertrophy Signaling	9.87E-02	4.02E-03	PRKAR1B
Colorectal Cancer Metastasis Signaling	1.06E-01	3.73E-03	PRKAR1B
G-Protein Coupled Receptor Signaling	1.13E-01	3.62E-03	PRKAR1B
Role of Macrophages, Fibroblasts and Endothelial Cells	1.32E-01	2.92E-03	PDGFA
Molecular Mechanisms of Cancer	1.47E-01	2.58E-03	PRKAR1B
Protein Kinase A Signaling	1.62E-01	2.46E-03	PRKAR1B
